# Large-scale evaluation of bacteriological-based method and qPCR performance for Brucellosis diagnosis in livestock using Bayesian latent class analysis

**DOI:** 10.1080/01652176.2025.2514753

**Published:** 2025-06-09

**Authors:** Giovanna Fusco, Alessandro Bellato, Lorena Cardillo, Agata Campione, Michela Di Roberto, Anna Cerrone, Francesca Bove, Roberta Pellicanò, Maria Ottaiano, Marco Esposito, Antonio Limone, Anna Rita Attili, Esterina De Carlo

**Affiliations:** a Department of Animal Health, Istituto Zooprofilattico Sperimentale del Mezzogiorno, Portici, Naples, Italy; b Department of Veterinary Sciences, Università di Torino, Grugliasco, Turin, Italy; c Regional Observatory of Epidemiology and Biostatistic, Istituto Zooprofilattico Sperimentale del Mezzogiorno, Portici, Naples, Italy; d UOD Prevenzione e Sanità Pubblica Veterinaria, Regione Campania, Naples, Italy; e General Direction, Istituto Zooprofilattico Sperimentale del Mezzogiorno, Portici, Naples, Italy; f School of Biosciences and Veterinary Medicine, Università di Camerino, Matelica, Macerata, Italy; g Scientific Direction, Istituto Zooprofilattico Sperimentale del Mezzogiorno, Portici, Naples, Italy

**Keywords:** Brucellosis, Bayesian modelling, bacteriological culture, sensitivity, specificity, livestock

## Abstract

The performance of direct tests, such as bacteriological culture and qPCR, for the diagnosis of brucellosis has been evaluated in a limited number of studies, often based on small sample sizes. Moreover, the absence of a gold standard makes this assessment even more challenging. A potential alternative for evaluating the performance of direct tests is Bayesian latent class analysis (BLCA), which does not require prior knowledge of disease status or a gold standard. This study aimed to estimate the sensitivity (Se) and specificity (Sp) of bacteriological culture for brucellosis diagnosis. In a brucellosis-endemic area, a large number of seronegative and seropositive buffaloes and cattle were tested using bacteriological culture and qPCR. BLCA was applied to estimate the performance of both tests. The median Se of bacteriological culture was estimated at 61.3%, compared to 70.9% of qPCR. The median Sp was 99.6% for bacteriological culture and 89.3% for qPCR. Bacteriological culture demonstrated a higher Positive Predictive Value (PPV) than qPCR in both buffaloes and cattle, whereas the Negative Predictive Value (NPV) of the two methods did not differ significantly. These results suggest that, in settings of low brucellosis prevalence, a positive bacteriological culture has a greater predictive value than qPCR .

## Introduction

Brucellosis is a widespread zoonotic disease affecting cattle, sheep, goats, pigs, several wild animals, and humans. It is caused by bacteria of the *Brucella* genus, which comprises 13 different species, each with distinct host preference, pathogenicity, and epidemiology. However, interspecies infections can occur when different host species share grazing areas or water sources (de Figueiredo et al. 2015; De Massis et al. 2019; Almuzaini 2023). Despite efforts to control or eradicate brucellosis, it remains a neglected zoonosis, affecting more than 170 countries worldwide. The estimated annual global incidence of 2.1 million cases is significantly higher than previously reported (Tao et al. 2021; Laine et al. 2023). In several Mediterranean, Middle Eastern, African, and Latin America countries, as well as in certain areas of Asia, the disease remains endemic, with a high prevalence in humans. *Brucella abortus*, *Brucella melitensis*, and *Brucella suis* are the species of greatest public health concern (Zhang et al. 2018; Food and Agriculture Organization of the United Nations 2013; World Organisation for Animal Health (WOAH) 2022). In these areas, human infection typically occurs through the consumption of contaminated raw milk and dairy products, as well as direct contact with body fluids of infected animals, particularly among people working with livestock (Laine et al. 2023; World Organisation for Animal Health (WOAH) 2023; Njeru et al. 2016). As a result, areas with endemic livestock brucellosis often overlap with regions reporting locally acquired human cases. This highlights the critical need for implementing effective control and eradication strategies in animals to reduce the incidence of human brucellosis, too (Dadar et al. 2021; European Health and Digital Executive Agency (HaDEA) 2023).

In livestock, brucellosis causes late-term abortion, placental retention, endometritis, and stillbirth, as well as orchitis and bursitis in males. Chronic infections can also lead to hygromas of the joints (Tulu 2022; European Food Safety Authority (EFSA), European Centre for Disease Prevention and Control (ECDC) 2024). Consequently, affected herds experience significant economic losses due to test-based culling measures, infertility, animal movement restrictions, vaccination campaigns, and facility disinfections requirements (European Health and Digital Executive Agency (HaDEA) 2023). Nevertheless, diagnosing brucellosis remains challenging. In livestock, it is primarily performed using *in vivo* serological tests, which indicate exposure to *Brucella* species. Commonly used serological tests include serum agglutination test (SAT), complement fixation test (CFT), indirect (I-) and competitive (c-) enzyme-linked immunosorbent assays (ELISA), and fluorescence polarization assay (FPA). These methods are widely applied to assess herd-level infection prevalence for surveillance purposes. However, no single serological test is fully adequate for all animal species or universally suitable across different epidemiological contexts. Therefore, combining multiple tests is necessary to confirm the diagnosis (World Organisation for Animal Health (WOAH) 2023). Notably, the SAT/CFT combination, which represents the most commonly used serial testing scheme, has shown limitation in specificity, as it fails to differentiate *Brucella*-infected animals from those with false-positive serological cross-reactions (Mainar-Jaime et al. 2005). A non-invasive alternative is the milk I-ELISA, which detects anti-*Brucella* antibodies in bulk milk and is effective for screening and monitoring dairy cattle. Recently, a promising antigen capture ELISA (Ag-cELISA) has been developed to detect both live and dead *Brucella* bacteria in milk samples (G¨urbilek et al. 2023). For the postmortem diagnosis, direct tests are used to detect *Brucella* organisms, including acid-fast staining, bacterial isolation, and polymerase chain reaction (PCR), followed by identification and typing methods (World Organisation for Animal Health (WOAH) 2023).

In Europe, a compulsory eradication program targets *B. abortus*, *B. melitensis*, and *B. suis* in livestock. To date, 21 European countries have granted brucellosis-free status for cattle population. However, the disease persists in Greece, Portugal, Italy, Bulgaria, and Hungary, although with a declining incidence (World Organisation for Animal Health (WOAH) 2023; European Health and Digital Executive Agency (HaDEA) 2023; European Food Safety Authority (EFSA) 2024). The reported herd prevalence in Europe decreased from 0.39% in 2023 to 0.25% in 2024 (European Food Safety Authority (EFSA), 2024). In Italy, the overall prevalence and incidence of brucellosis in cattle and buffalo populations were 0.16 and 0.12%, respectively, in 2023. While most provinces in Northern and Central Italy have achieved the brucellosis-free status, the disease remains endemic in some regions of Southern Italy, including in Campania (Sistema Informativo Veterinario (VetInfo) 2024). Out of the five provinces in Campania region, three are brucellosis-free – Avellino, Benevento and Naples –, in Salerno province brucellosis was present with a 0.5% prevalence in the cattle population in 2022, whereas in Caserta the disease was recorded with 13.26% prevalence in both cattle and buffaloes (Sistema Informativo Veterinario (VetInfo) 2024). To address this, stricter regional measures have been implemented to accelerate eradication efforts in cattle and buffaloes. For instance, at slaughter, tissue samples are collected not only from seropositive animals but also from seronegative animals originating from suspect or confirmed infected herds. These samples undergo bacteriological culture and/or real-time PCR (qPCR) for disease confirmation (Regional deliberation n. 104 of March 8th, 2022).

According to the European, national, and regional regulations, the official method for diagnosing *Brucella* spp. is direct bacteriological culture. However, its sensitivity is reported to be relatively low, as field samples often contain limited bacterial loads (European Union Reference Laboratory (EURL) for Brucellosis 2021). Moreover, the true sensitivity and specificity of this assay remain poorly defined (O’Grady et al. 2014; Rodrigues Dos Santos Souza et al. 2022). In the absence of a gold standard for comparison, assessing the accuracy of a diagnostic test is inherently challenging. To overcome this limitation, several authors (Johnson et al. 2019; Rodrigues Dos Santos Souza et al. 2022) and the World Organization for Animal Health (WOAH) recommended the use of Bayesian Latent Class Analysis (BLCA) (World Organisation for Animal Health (WOAH), 2022). BLCA assumes that no diagnostic test is perfect and estimates sensitivity and specificity based on the joint results of multiple tests (Johnson et al. 2019; World Organisation for Animal Health (WOAH) 2023).

This study aimed to evaluate the sensitivity and specificity of bacteriological culture and qPCR by applying BLCA to the results obtained from samples collected by official veterinary services as part of the brucellosis eradication program over a two-year period (2022–2023).

## Materials and methods

### Adherence to guidelines

This study adhered to the STARD-BLCM guidelines for reporting the design, conduct and results of diagnostic accuracy studies using BLCA models (Supplementary Table 1) (Kostoulas et al. 2017).

All diagnostic procedures followed validated and accredited methodologies, in accordance with the WOAH Terrestrial Manual and the guidelines of the European Union Reference Laboratory (EURL) for Brucellosis (World Organisation for Animal Health (WOAH) 2023; European Union Reference Laboratory (EURL) for Brucellosis 2021a, 2021b).

### Study design and population

This retrospective study analysed data collected within the framework of the brucellosis eradication program in Campania. The dataset comprised results from serological, bacteriological, and molecular tests. Data from the province of Caserta and Salerno were included in the analysis, as brucellosis has been eradicated from the other three provinces of Campania (Sistema Informativo Veterinario (VetInfo) 2024).

In accordance with regulatory requirements, local veterinary authorities conducted *in vivo* serological testing (i.e. SAT and CFT) as part of the brucellosis eradication campaign in cattle and buffalo farms. Based on the test results, 157 farms were classified as either confirmed or suspect infected. In 2022, 5,149 animals were slaughtered in the provinces of Caserta and Salerno originating from those farms. They included 4,902 buffaloes (95.2%) and 247 cattle (4.8%). Of them, 3,838 animals (74.5%) were compulsorily culled following a positive *in vivo* serological test (SAT and CFT) during the eradication campaign. Among the 1,311 animals (25.5%) that tested negative by *in vivo* serological examinations, 114 buffaloes were mandatorily culled as part of stamping-out measures, while 1,197 (125 cattle and 1,072 buffaloes) were voluntarily culled. At slaughter, all 5,149 animals were sampled to confirm *Brucella* spp. infection using direct tests, namely, bacteriological culture and qPCR.

### Sample collection

At slaughter, the local veterinary health authorities collected specimens during postmortem inspection. The specimens included head, mammary, and genital lymph nodes, spleen, pregnant or early post-partum uterus, testis, udder, amniotic fluid, and foetal membranes. Each specimen was placed in triple wrapping and immediately transported under refrigeration (5 ± 3 °C) to the laboratory of the Istituto Zooprofilattico Sperimentale del Mezzogiorno (IZSM) in Portici (Southern Italy). Specimens that were not promptly delivered to the laboratory or immediately cultured were frozen at ≤ −18 °C.

### Collection of tissue samples in the necropsy room

In the necropsy room, trained personnel aseptically removed the specimens from their packaging and prepared them by excising unnecessary material (e.g. fat). To minimize contamination, the exposed surfaces were cauterized using a spatula heated on a Bunsen burner before incision. A sterile scalpel was then used to make an incision, and a sterile flocked swab (COPAN Diagnostic Inc., Brescia, Italy) was rubbed across the entire exposed internal surface where the incision was made. The swab was then placed back into its container and transported to a Biosafety Level-3 (BSL-3) laboratory for parallel bacteriological culture and qPCR testing.

The collection method was validated by comparing three different sample processing techniques: maceration, grounding, and flocked swab collection. This validation, conducted in three independent parallel working sessions, led to the identification of five additional positive samples compared to the standard technique (data not shown).

### Bacteriological analyses

The bacteriological identification was carried out in the BSL-3 laboratory using validated and accredited procedures with small modifications (World Organisation for Animal Health (WOAH) 2023), followed by identification and typing according to the standard operating procedures provided by the European Union Reference Laboratory (EURL) for brucellosis (European Union Reference Laboratory (EURL) for Brucellosis 2021c). Briefly, the flocked swabs from the necropsy room were immediately streaked onto Brucella agar and/or CITA agar plates, incubated at 37 °C ± 1 °C in 5–10% CO2 atmosphere and routinely examined up to 10 days. At least three colonies presumed to be *Brucella* spp. were subcultured on blood agar or brain-heart infusion (BHI) agar plates and incubated at 37 °C ± 1 °C in 5–10% CO_2_ atmosphere for 48–72 h. Next, the colonies underwent identification and biotyping tests, through Gram staining, urease, oxidase, agglutination test using anti-S-Brucella polyclonal serum (ZM01/R30164801-Thermo Fisher Scientific, Waltham, Massachusetts, US), phage lysis and dye susceptibility, as well as miniaturised biochemical procedures, with VITEK 2 Compact system (bioMérieux, Lyon, France). Next, the developed colonies were submitted to real time PCR for the detection of *Brucella* spp. (as described below) followed by species and Biovar identification, by the National Reference Centre for Brucellosis (NRCB), Istituto Zooprofilattico Sperimentale di Abruzzo e Molise (IZSAM).

### Biomolecular examinations

Swabs were first thermally inactivated at 99 °C for 10 min and transferred to 2 mL tubes with the addition of 200 μm in diameter glass beads (NextAdvance, Troy, NY, USA). Samples were suspended in 1 mL sterile Phosphate-Buffer Saline (PBS) and incubated at room temperature for 2 h on magnetic stirrer. Afterwards, samples were vortexed and mechanically homogenized using TissueLyser (Qiagen, Hilden, Germany) for 5 min at 30 Hz and clarified by brief centrifugation at 6800 × g (Eppendorf, Hamburg, Germany). Aliquots of 200 μL of supernatant were collected for nucleic acids extraction and purification, using QIAsymphony automated system (Qiagen, Hilden, Germany) with QIAsymphony DSP Virus/Pathogen Mini Kit (Qiagen). During the DNA extraction step, an internal control specific for the PCR amplification kit (QIAGEN, Hilden, Germany) was added to all samples. Extracted DNA was eluted in 60 μL Elution Buffer and stored at −20 °C until further analyses.

*Brucella* spp. was detected according to the protocol reported by the EURL for brucellosis (European Union Reference Laboratory (EURL) for Brucellosis 2021a), by real-time PCR (qPCR) targeting only the Insertion Sequence (IS) 711, which is specific and up to 10-fold more sensitive than *bcsp31* or *per* targets (Bounaadja et al. 2009).

Each reaction mixture contained QuantiFast Pathogen Master Mix at 1X final concentration (Qiagen), Internal Control Assay (Qiagen) at 1X final concentration, 0.5 μM of each primer, 0.2 μM of probe, 5 μL of DNA and 9.5 μL of nuclease-free water in a final volume of 25 μL. The PCR thermal profile was as follows: initial template denaturation at 95 °C for 5 min, and 45 cycles including a template denaturation step at 95 °C for 15 s followed by a primer annealing/extension step at 60 °C for 30 s. The assays were performed on the real-time thermal cycler CFX96 Touch Real-Time PCR Detection System (Bio-Rad Laboratories, Hercules, CA, USA) in the presence of a negative control (purified PCR-grade water), a negative extraction control (all reagents used for DNA extraction) and a positive control (*B. abortus* genomic DNA).

### Estimation of test sensitivity and specificity

In the absence of a true gold standard method, the Hui-Walter model for BLCA (Hui and Walter 1980) was applied to estimate the sensitivity and specificity of both bacteriological culture and qPCR. The model assumes that both qPCR and bacteriological culture have uncertain sensitivity and specificity. Therefore, neither can be used as a reference standard.

Our sample consisted not of animals drawn from the general population but of slaughtered animals from confirmed or suspect infected farms. About the animals, we had prior knowledge of their serological status. Therefore, we identified four distinct populations, each expected to have different prevalence rates (*Prev*𝑗). The populations were: (*j* = 1) seronegative cattle, (*j* = 2) seronegative buffaloes, (*j* = 3) seropositive cattle, and (*j* = 4) seropositive buffaloes. The latter two populations (*j* = 3, 4) comprised animals slaughtered due to their seropositive status, while the first two (*j* = 1, 2) included those culled for stamping out measures or other reasons. Each farm could host animals belonging to one or more of the *j* populations, resulting in *k* population-farm combinations (herds). Each *k* combination has its own prevalence, which was modelled as follow:

logit(Prevk)=logit Preevj+ δk

where 𝛿*k* was modelled as 𝛿*k*∼*N*(0, 𝜎2), and 𝜎∼*Half Cauc*ℎ*y*(0,5).

Having two tests (T1 and T2), we observed *t* = 4 distinct combinations of results: T1− and T2− (*t* = 00); T1− and T2+ (*t* = 01); T1+ and T2− (*t* = 10); and T1+ and T2+ (*t* = 11).

The data for herd *k* are given by the vector *yk* = (*y*00,*k*,*y*01,*k*,*y*10,*k*,*y*11,*k*), where *yt*,*k* is the number of animals from herd *k* testing the *t* combination. The independent vectors *y*1,*y*2,…,*yk* constituted the complete data set. The data were assumed to have independent multinomial distributions:

yk∼Multinomial(Probt,k,Nk)

where *Nk* is the number of animals from the *k*th herd, and *Probt*,*k* = *Set*,*k* + *Spt*,*k*. The probability of observing a given *t* combination depends on the *k*th herd prevalence, sensitivities (*SeT*1, *SeT*2), specificities (*SpT*1, *SpT*2), and covariance terms (*Cov*(*SeT*1,*SeT*2), *Cov*(*SpT*1,*SpT*2)) capturing conditional dependence between tests. Therefore, *Probt*,*k* is defined as:

$$\begin{array}{l} \text{Prob}00,k=\text{Prevk}\left(\left(1\text{SeT}1\right)\left(1\text{SeT}2\right)\right)+\text{cov}\left(\text{SeT}1,\;\text{SeT}2\right)\\ \;+\left(1\text{Prevk}\right)\left(\text{SpT}\;1\text{SpT}2+\text{cov}\left(\text{SpT}1,\text{SpT}2\right)\right) \end{array}$$

$$\begin{array}{l} \text{Prob}01,k=\text{Prevk}\left(\left(1\text{SeT}1\right)\text{SeT}2+\text{Cov}\left(\text{SeT}1,\;\text{SeT}2\right)\right)+\left(1\text{Prevk}\right)\left(\text{SpT}1\left(1-\text{SpT}2\right)+\text{Cov}\left(\text{SpT}1,\text{SpT}2\right)\;\right)\\ \text{Prob}10,k=\text{Prevk}\left(\text{SeT}1\left(1\text{SeT}2\right)+\text{Cov}\left(\text{SeT}1,\;\text{SeT}2\right)\right)+\left(1\text{Prevk}\right)\left(1-\text{SpT}\;1\right)\text{SpT}\;2\\ +\text{Cov}\left(\text{SpT}1,\text{SpT}2\right)) \end{array}$$

$$\begin{array}{l} \text{Prob}11,k=\text{Prevk}(\text{SeT}1,\;\text{SeT}2+\text{cov}\left(\text{SeT}1,\;\text{SeT}2\right)\\ \;+\left(1\text{Prevk}\right)\left(\left(1\text{SpT}1\right)\left(1\text{SpT}2\right)+\text{cov}\left(\text{SpT}1,\text{SpT}2\right)\right) \end{array}$$

where *SeT*1 and *SeT*2 are the sensitivities and *SpT*1 and *SpT*2 are the specificities of the two tests.

To model a possible conditional dependence between the two diagnostic tests, we included covariance terms *Cov*(*SeT*1, *SeT*2) and *Cov*(*SpT*1,*SpT*2) as described by Branscum et al. (2004):

Cov(SeT1, SeT2)=Prob T1+T2+|D++SeT1 SeT2

Cov(SpT1, SpT2)=Prob T1−T2−|D−+SpT1SpT2

where *Prob T*1+*T*2+│*D*+ was the probability of both tests being positive, given the presence of disease, and *Prob T*1―*T*2―│*D*― was the probability of both tests being negative given the absence of disease. Since prior information about the two covariances is unavailable, uniform prior distributions over the ranges described by Dendukuri and Joseph (2001) were modelled:

$$\begin{array}{l} \text{cov}\left(\text{SeT}1,\text{SeT}2\right)∼\\ \text{Uniform}\left[\left(\text{SeT}11\right)\left(1\text{SeT}2\right),\text{min}\left(\text{SeT}1,\text{SeT}2\right)\text{SeT}1\text{SeT}2\right] \end{array}$$

$$\begin{array}{l} \text{cov}\left(\text{SpT}1,\text{SpT}2\right)∼\\ \text{Uniform}\left[\left(\text{SpT}11\right)\left(1\text{SpT}2\right),\text{min}\left(\text{SpT}1,\text{SpT}2\right)\text{SpT}1\text{SpT}2\right] \end{array}$$

The model was run in JAGS version 4.3.1, calling it from R environment (R version 4.4.3, 2025-02-28 ucrt) through the *runjags* package (Denwood 2016; Plummer 2023). The simulation included two MCMC chains of 500,000 iterations each, with 5,000 iterations discarded as burn-in for adaptation. A total of 50,000 samples were retained using a thinning factor of 10 to reduce autocorrelation, employing the Metropolis-Hastings algorithm.

We derived the estimates from the distribution of Monte Carlo Markov Chain (MCMC) simulated values, with results expressed as the median and the 95% Credibility Interval (95% CrI).

From the estimates of test sensitivity and specificity, the Positive Predictive Value (PPV) and the Negative Predictive Value (NPV) were calculated for both *T* test in every kth herd:

$$\text{PPV}=\frac{\text{Se}*\text{Prev}}{\left(\text{Se}*\text{Prev})+(\left(1-\text{Sp})*(1-\text{Prev}\right)\right)}$$

$$\text{NPV}=\frac{\text{Sp}*(1-\text{Prev})}{\left(\text{Sp}*(1-\text{Prev}))+((1-\text{Se})*\text{Prev}\right)}$$

The analysis was carried out using R statistical software version 4.3.1 (R Core Team, A Language and Environment for Statistical Computing. R Foundation for Statistical Computing, Vienna, Austria. https://www.R-project.org).

The sensitivity and specificity of the bacteriological culture were set *a priori* as a *SeT*1 ∼ *Beta*(9.53, 8.71) and *SpT*1 ∼ *Beta*(16.34, 0.98), which corresponded to 52.2% sensitivity (95% Confidence Interval [95% CI]: 30.0 − 74.1%) and 94.3% specificity (95% CI: 80.0 − 99.9%). That was based on a review of diagnostic tools for brucellosis diagnosis in cattle (Gall and Nielsen 2004) and two comparative studies (O’Grady et al. 2014; Hosein et al. 2017) suggesting that the sensitivity of bacteriological culture performed with standard WOAH method range between 46 and 62%, while the specificity is generally considered as high as almost 100%. Similarly, based on a review (Mabe et al. 2022) and a comparative study (Abdel-Hamid et al. 2021), the sensitivity and specificity of the qPCR assay were *a priori* set to *SeT*2 ∼ *Beta*(18.01, 1.17), and *SpT*2 ∼ *Beta*(26.55, 3.48), corresponding to 93.9% sensitivity (95% CI: 80.0 − 99.7%) and 88.4% specificity (95% CI: 75.0 − 97.0%).

The *a priori* prevalence of infection in seronegative animals was assumed alike that recorded in the same region in 2021, namely, *Prev*𝑗 = 1 ∼ *Beta*(0.52, 50.05) and *Prev*𝑗 = 2 ∼ *Beta*(5.48, 35.84), which corresponded to a median expected prevalence of 0.1% in cattle and 12.7% in buffaloes.

Although a higher prevalence was expected, there was no information available for seropositive animals. Therefore, we set uninformative priors, *Prev*𝑗 = 3,4 ∼  *Uniform*[0,1], for the prevalence of infection in seropositive cattle and buffaloes.

To assess the impact of prior information on posterior estimates and how assumptions influence diagnostic performance metrics, a sensitivity analysis was performed testing a total of 14 scenarios. We tested combinations of uninformative priors, ∼ *Uniform*[0,1], for tests’ sensitivity and specificity and different expected prevalence, from low (0.1%) to high (25%) in both species.

### Model diagnostics

The accuracy of the simulations was assessed checking that the Monte Carlo error (MCerr) was lower than 5% of the standard deviation of the estimate. The independence of the sampling was measured through effective sample size (SSeff) and autocorrelation at lag 100 (AC100). The potential scale reduction factor, calculated by the Gelman-Rubin diagnostics (*R*), was used to assess the convergence of the MCMC sampling. Values of *R* close to 1 for all monitored parameters indicated good mixing of the chains and convergence, supporting the reliability of the posterior estimates. In addition, posterior predictive checks (PPC) were performed by generating simulated datasets from the posterior distributions of the model parameters and comparing key summary statistics of the simulated data with those of the observed data to ensure the model captured the underlying data structure.

## Results

### Bacteriological and biomolecular results

Out of 5,149 animals, 1,869 (36.3%) tested positive for at least one of the two direct techniques for *Brucella* detection. Among them, 501 (26.8%) tested positive to both tests, 937 (50.1%) tested positive to qPCR only, and 431 (23.1%) tested positive to bacteriological culture only. Overall, the qPCR detected a greater number of positives than the bacteriological culture. On the other hand, bacteriological culture detected 80 animals, which not only tested negative to qPCR but also to the serological tests.

Among the 1,311 seronegative animals, 24.3% (*n* = 318) tested positive to at least one of the two direct methods (Table 1).

**Table 1.** Results of serological and direct tests.

		qPCR				
		Cattle		Buffaloes		
Serology (SAT & CFT)	Bacteriological culture	Neg	Pos	Neg	Pos	Total
Neg	Neg	100	23	893	141	1,157
	Pos	1	1	73	79	154
Pos	Neg	54	25	2,233	748	3,060
	Pos	22	21	335	400	778
	Total	177	70	3,534	1,368	5,149

The table diplays the results of parallel serological tests performed in the framework of the eradication campaign compared with the results of direct tests (bacteriological culture and qPCR) performed at slaughter.

Among 125 seronegative cattle (*j = 1*), 0.8% tested positive to both tests, 0.8% tested positive to bacteriological culture only, and 18.4% tested positive to qPCR only. Among 1,186 seronegative buffaloes (*j* = 2), 6.7% tested positive to both tests, 6.2% tested positive to bacteriological culture only, and 11.9% tested positive to qPCR only. Among 122 seropositive cattle (*j* = 3), 17.2% tested positive to both tests, 18.0% tested positive to bacteriological culture only, and 20.5% tested positive to qPCR only. Eventually, among 3,716 seropositive buffaloes, 10.8% tested positive to both tests, 9.0% tested positive to bacteriological culture only, and 20.1% tested positive to qPCR only (Sistema Informativo Veterinario (VetInfo) 2024).

Sℎould tℎe bacteriological culture and qPCR *be used in* series (*T*1 ∧ *T*2) it would result in 6.1% positive results among seronegative animals, and 11.0% positive results among seropositive animals. On the other hand, if bacteriological culture and qPCR were used in parallel (*T*1 ∨ *T*2), they would provide 24.3 and 40.4% positive results in seronegative and seropositive animals, respectively. For seronegative animals, the use of test in series would result in 74.9% less positives compared to parallel use. For seropositive animals, the use of tests in series would reduce the number of positive results by 72.9% (Supplementary Tables 2 and 3).

**Table 2.** Results of the Bayesian latent class analysis model for estimating test sensitivity and specificity with four populations.

Parameter	Median (%)	Mean ± S.D.	95% CrI	SSeff
Bact. culture Se	61.3	61.3 ± 2.1%	57.1–65.4%	37,447
Bact. culture Sp	99.6	99.5 ± 0.4%	98.7–100%	32,431
qPCR Se	71.0	70.9 ± 1.7%	67.4–74.1%	39,909
qPCR Sp	89.3	89.3 ± 1.0%	87.2–91.3%	34,601
Prev._cattle sero−_	0.8	1.2 ± 1.3%	0.0–3.8%	38,799
Prev._buffaloes sero−_	6.6	6.8 ± 1.9%	3.4–10.5%	24,605
Prev._cattle sero+_	30.8	31.9 ± 12.1%	9.5–55.4%	26,946
Prev._buffaloes sero+_	28.3	28.4 ± 4.8%	19.2–37.9%	10,468
Cov (Se_1_, Se_2_)	−10.0	−9.8 ± 0.9%	−11.4 to 8.0%	37,180
Cov (Sp_1_, Sp_2_)	0.0	0.1 ± 0.1%	−0.1 to 0.3%	38,780

S.D.: standard deviation; 95%CrI: 95% credibility interval; SSeff: effective sample size; Se: sensitivity; Sp: specificity.

**Table 3.** Results of the Bayesian latent class analysis model for estimating positive and negative predictive values (PPV and NPV, respectively) for both bacteriological culture and qPCR in the general population of cattle and buffaloes in the province of caserta, Southern Italy.

Parameter	Population	Serology result	Median (%)	95% CrI
PPV_bact_	Cattle	−	54.8	0.0–100%
PPV_bact_	Buffalo	−	91.0	61.0–100%
PPV_bact_	Cattle	+	98.4	82.5–100%
PPV_bact_	Buffalo	+	98.2	91.4–100%
NPV_bact_	Cattle	−	99.7	98.6–100%
NPV_bact_	Buffalo	−	97.3	96.1 − 98.5%
NPV_bact_	Cattle	+	85.2	70.0 − 95.6%
NPV_bact_	Buffalo	+	86.7	82.6 − 90.6%
PPV_qPCR_	Cattle	−	5.4	0.0 − 25.4%
PPV_qPCR_	Buffalo	−	32.1	15.6 − 50.1%
PPV_qPCR_	Cattle	+	74.8	35.8 − 91.4%
PPV_qPCR_	Buffalo	+	72.4	55.7 − 83.9%
NPV_qPCR_	Cattle	−	99.7	98.9–100%
NPV_qPCR_	Buffalo	−	97.7	96.8 − 98.7%
NPV_qPCR_	Cattle	+	87.4	74.0 − 96.2%
NPV_qPCR_	Buffalo	+	88.7	85.3 − 91.8%

S.D.: standard deviation; 95% CrI: 95% credibility interval; SSeff: effective sample size.

### Model diagnostics

The complete simulation model was specified based on 5,149 observations and 219 herds. The convergence was immediately reached for all observed parameters after burn-in simulations. MCMC displayed near-perfect convergence, as evidenced by *R* consistently below 1.01, SSeff sufficiently large (>10,000) for all monitored parameters, and AC100 approaching zero. MCerr was negligible across all parameters (<1.0%) and PPC confirmed that simulated data had distributions comparable with observed data (Supplementary Table 4).

### Sensitivity analysis

The priors selected had a limited impact on parameter estimates. In particular, uninformative priors decreased the estimated sensitivity of both tests, by (average modulo ± standard deviation) 5.5 ± 3.7% for bacteriological culture, and by 6.7 ± 5.3% for qPCR. On the other hand, uninformative priors had limited to none effect on test specificity, with a variation of 1.0 ± 0.9% for bacteriological culture, and 1.6 ± 0.8% for qPCR. The selection of prevalence priors among uninformative, low (0.1%), and high (25.0%) alternatively in cattle and buffaloes, led to a bacteriological culture sensitivity variation of 5.7 ± 2.7%, and 4.7 ± 2.4% for specificity. Regarding qPCR, the variation was 3.0 ± 2.8% for sensitivity, and 6.3 ± 2.5% for specificity. The widest variations were observed selecting the most inappropriate prior combination with high prevalence of brucellosis in cattle and low in buffaloes.

### Estimated prevalence

Since the data were not collected from a global population, it was not possible to estimate the true prevalence through the model. Instead, the model estimated the proportion of infected cattle and buffaloes depending on their serological status. Seronegative cattle (*j* = 1), which were slaughtered for other reasons than being seropositive, had the lowest proportion of infection (0.8%, 95% CrI: 0.0 − 3.8%) (Table 2). Similarly, seronegative buffaloes (*j* = 2), also slaughtered for other reasons than being seropositive, had a 6.6% proportion (95% CrI: 3.4 − 10.5%). Among seropositive cattle (*j* = 3), the estimated proportion of infection was 30.8% (95% CrI: 9.6 − 55.4%). Eventually, the infection proportion in seropositive buffaloes (*j* = 4) was 28.3% (95% CrI: 19.2 − 37.9%). For each herd, the model estimated a posterior prevalence; they ranged between 0.5 and 98.3% (mean = 22.8%).

### Estimation of test sensitivity and specificity

The estimated median sensitivity of the bacteriological culture was 61.3% (95% CrI: 57.1 − 65.4%) with a median specificity of 99.6% (95% CrI: 98.7–100%). For qPCR, the estimated median sensitivity was 71.0% (95%CrI: 67.4 − 74.1%) with a median specificity of 89.3% (95% CrI: 87.2 − 91.3%) (Table 2).

The PPV and the NPV varied across all herds depending on herd-level prevalence, based on test performance. For bacteriological culture, the PPV was estimated 90.6% (95% CrI: 54.9 − 99.9%) and the NPV was 86.9% (95% CrI: 67.2 − 95.3%). For qPCR, the PPV was 49.8% (95% CrI: 24.1 − 80.3%), and the NPV was 88.3% (95% CrI: 69.9 − 95.9%). Aggregated PPV and NPV are reported in Table 3.

## Discussion

Bovine brucellosis persists in Italy despite a national eradication program initiated nearly 60 years ago. Outbreaks continue to occur in southern regions, such as Campania, with buffalo farms in the province of Caserta being especially affected. Although the WOAH recommends bacterial culture for outbreak confirmation due to its high specificity and designates it as the standard for confirming clinical or suspected cases (World Organisation for Animal Health (WOAH) 2023; Chisi et al. 2017), this method has notable limitations. Specifically, bacteriological culture may yield false negatives in samples with low bacterial loads, and it is both time-consuming and poses biosafety risks due to potential laboratory-acquired infections (World Organisation for Animal Health (WOAH) 2023; European Union Reference Laboratory (EURL) for Brucellosis 2021b; Liu et al. 2023). In addition, while bacteriological culture was once the only confirmatory test, advances in diagnostic technology have led to the acceptance of alternative methods, such as molecular techniques, which is now recognized under current European and Italian regulations (Commission Delegated Regulation (EU) 2019; DECRETO 2 maggio 2024). However, this test is also imperfect, with issues such as interference from inhibitors or sample degradation affecting qPCR performance (World Organisation for Animal Health (WOAH) 2023). Consequently, several authors have underscored the need to employ multiple diagnostic methods (Mahajan et al. 2017).

Given these challenges, our study employed Bayesian latent class analysis (BLCA) to jointly estimate the sensitivity and specificity of bacterial culture and qPCR (World Organisation for Animal Health (WOAH) 2023; Hui and Zhou 1998). Our analysis was based on an extensive dataset collected as part of the national brucellosis eradication program. It is important to note that our data do not originate from random sampling of the general population; instead, they derive from confirmed or suspected infected farms. This precludes estimation of the true population prevalence but provides a robust sample of cases processed under official protocols.

To account for the hierarchical structure inherent in the data—comprising cattle and buffaloes, both seropositive and seronegative, with variable herd-level prevalence—we estimated prevalence for each herd. This approach allowed us to calculate average positive and negative predictive values for the diagnostic tests.

In our model, we incorporated prior information on the expected prevalence in the general population, which, in our study, was approximated by data from seronegative animals. It appears that our priors may have overestimated the prevalence in these animals, as indicated by the posterior estimates. However, given the nature of the data, which derived from confirmed or suspect infected herds only, the estimates cannot be generalized to the general populations of cattle and buffaloes of the Campania region. In contrast, we adopted non-informative priors for seropositive animals, since the relationship between serological and direct test outcomes remains unclear. Although we anticipated a higher prevalence among these animals, the exact magnitude was uncertain. Indeed, we observed posterior estimates higher than in seronegative animals.

The sensitivity and specificity parameters for both tests were derived from the literature, although available studies are limited and report discordant values. The estimated sensitivity and specificity of the bacteriological culture were demonstrated to be in line with or slightly higher than those previously described (O’Grady et al. 2014; Hosein et al. 2017), while in another study lower specificity (55%) were observed, compared to our results (Nyarku et al. 2020). Consistently, the estimated sensitivity of the qPCR was in line with that previously described (Abdel-Hamid et al. 2021).

A sensitivity analysis was conducted to confirm the robustness of our estimates; the results showed only minor fluctuations when uninformative priors were employed. More pronounced variability, we observed posterior estimates higher than in seronegative animals.

The sensitivity and specificity parameters for both tests were derived from the literature, although available studies are limited and report discordant values. The estimated sensitivity and specificity of the bacteriological culture were demonstrated to be in line with or slightly higher than those previously described (O’Grady et al. 2014; Hosein et al. 2017), while in another study lower specificity (55%) were observed, compared to our results (Nyarku et al. 2020). Consistently, the estimated sensitivity of the qPCR was in line with that previously described (Abdel-Hamid et al. 2021).

A sensitivity analysis was conducted to confirm the robustness of our estimates; the results showed only minor fluctuations when uninformative priors were employed. More pronounced variability by prior assumptions. Regarding disease prevalence, uninformative priors had minimal impact across all populations, but strong prior assumptions, particularly in seronegative animals, led to modest shifts in posterior prevalence, with the most notable changes observed when assuming divergent prevalence levels between cattle and buffaloes.

To consider a possible conditional dependence between the two diagnostic tests, we included covariance terms. The negative covariance between sensitivities suggests that when one test yields a false negative, the other is more likely to be correct. This was consistent with tests targeting different bacterial components. On the other hand, the near-zero covariance for specificities aligns with conditional independence given true negatives.

In addition to estimating sensitivity and specificity, we also assessed the predictive values of both tests under varying prevalence conditions. As expected, the positive predictive value of both bacteriological culture and qPCR increased in high-prevalence settings and decreased in low-prevalence populations, while the negative predictive value followed the opposite trend. Although the use of enrichment media—particularly recommended for milk, blood, other body fluids, and certain tissue samples—can enhance the performance of bacteriological culture (World Organisation for Animal Health (WOAH) 2023), this method is still considered less sensitive due to limitations related to suboptimal sample storage and low bacterial loads. Nevertheless, our results consistently showed higher PPV values for bacteriological culture compared to qPCR across all population groups. Notably, among seropositive animals, while the PPV of qPCR was estimated to be as low as 5%, the PPV of bacteriological culture exceeded 50%. This suggests that in low-prevalence contexts, a positive bacteriological culture result may be more reliable than a positive qPCR result.

However, due to the limited PPV associated with low prevalence, a positive result from either test should ideally be confirmed using the other (i.e. tests in series) to increase diagnostic confidence for Brucella spp. infection (Abernethy et al. 2012). Conversely, in high-prevalence settings such as seropositive subpopulations, a single positive result from either method may be sufficient to confirm infection, while negative results should be interpreted with caution and ideally confirmed by both tests (i.e. tests in parallel).

The implementation of stringent regional measures, particularly the use of direct detection methods at slaughterhouses and the sampling of seronegative animals from infected, suspect, or high-risk herds, has proven to be an effective tool for the early detection of *Brucella* spp. Indeed, some of the animals testing positive through direct methods had previously tested negative by serology in the months prior to slaughter. Our findings support earlier reports documenting that 16.5 to 22% of animals may yield positive culture results despite being seronegative according to different serological assays (Abernethy et al. 2012; O’Grady et al. 2014).

Several hypotheses could explain these results. One possibility is the presence of latent infections-well-documented in the literature (Lapraik and Moffat 1982; Ramírez-Romero 1998; O’Grady et al. 2014) – which can contribute to the re-introduction or persistence of brucellosis in officially disease-free herds (ter Huurne et al. 1993). Calves infected in utero or through colostrum may harbor persistent infections that are undetectable by serological tests, representing a substantial challenge to eradication efforts. Another possibility is that these animals were recently infected and had not yet developed detectable antibody responses (Corner et al. 1987; Zowghi et al. 1990), or that serological tests failed to detect antibodies due to inherent limitations in assay sensitivity, leading to false-negative results.

In conclusion, although our study has inherent limitations, its findings contribute to clarifying the performance of bacteriological culture and qPCR for brucellosis diagnosis. A logical next step would be to investigate bacteriological culture and qPCR performance at the level of individual organs to identify the most informative anatomical sites for analysis.

## Conclusion

Given the significant economic and public health implications of brucellosis outbreaks in livestock, prompt and accurate diagnosis is essential to ensure timely intervention and containment (Mahajan et al. 2017).

Although bacteriological culture is often regarded as less sensitive than molecular methods, both approaches have proven valuable in detecting *Brucella* infections in animals that tested negative using other diagnostic tools. Moreover, our large-scale analysis of diagnostic performance revealed that bacterial culture may exhibit a higher positive predictive value than qPCR under specific epidemiological conditions. These findings highlight that neither test is sufficient on its own and that complementary use of both is warranted. Diagnostic strategies should therefore be tailored to the context, considering test results as well as the underlying risk within the population.

## Supplementary Material

Supplementary_tables.docx

## Data Availability

All data supporting the results of the paper are included within the manuscript.

## References

[CIT0001] Abdel-Hamid NH, Beleta EIM, Kelany MA, Ismail RI, Shalaby NA, Khafagi MHM. 2021. Validation of real-time polymerase chain reaction versus conventional polymerase chain reaction for diagnosis of brucellosis in cattle sera. Vet World. 14(1):144–154. doi: 10.14202/vetworld.2021.144-154. 33642798 PMC7896886

[CIT0002] Abernethy DA, Menzies FD, McCullough SJ, McDowell SW, Burns KE, Watt R, Gordon AW, Greiner M, Pfeiffer DU. 2012. Field trial of six serological tests for bovine brucellosis. Vet J. 191(3):364–370. doi: 10.1016/j.tvjl.2011.03.008. 21550272

[CIT0003] Almuzaini AM. 2023. An epidemiological study of *Brucellosis* in different animal species from the Al-Qassim Region, Saudi Arabia. Vaccines. 11(3):694. doi: 10.3390/vaccines11030694. 36992277 PMC10056860

[CIT0004] Bounaadja L, Albert D, Chénais B, Hénault S, Zygmunt MS, Poliak S, Garin-Bastuji B. 2009. Real-time PCR for identification of *Brucella* spp.: a comparative study of IS711, bcsp31 and per target genes. Vet Microbiol. 137(1–2):156–164. doi: 10.1016/j.vetmic.2008.12.023. 19200666

[CIT0005] Branscum AJ, Gardner IA, Johnson WO. 2004. Bayesian modeling of animal- and herd-level prevalences. Prev Vet Med. 66(1–4):101–112. doi: 10.1016/j.prevetmed.2004.09.009. 15579338

[CIT0006] Chisi SL, Marageni Y, Naidoo P, Zulu G, Akol GW, Van Heerden H. 2017. An evaluation of serological tests in the diagnosis of bovine brucellosis in naturally infected cattle in KwaZulu-Natal province in South Africa. J S Afr Vet Assoc. 88(0):e1–e7. doi: 10.4102/jsava.v88i0.1381. PMC6138170 28281771

[CIT0007] Commission Delegated Regulation (EU) 2020/689 of 17 December 2019 supplementing Regulation (EU) 2016/429 of the European Parliament and of the Council as regards rules for surveillance, eradication programmes, and disease-free status for certain listed and emerging diseases. 2019. [accessed 2025 Mar 17]. https://eur-lex.europa.eu/legalcontent/EN/TXT/?uri=CELEX%3A02020R0689-20231011.

[CIT0008] Corner LA, Alton GG, Iyer H. 1987. Distribution of *Brucella abortus* in infected cattle. Aust Vet J. 64(8):241–244. doi: 10.1111/j.1751-0813.1987.tb09692.x. 3120683

[CIT0009] Dadar M, Tiwari R, Sharun K, Dhama K. 2021. Importance of Brucellosis control programs of livestock on the improvement of one health. Vet Q. 41(1):137–151. doi: 10.1080/01652176.2021.1894501. 33618618 PMC7946044

[CIT0010] de Figueiredo P, Ficht TA, Rice-Ficht A, Rossetti CA, Adams LG. 2015. Pathogenesis and immunobiology of brucellosis: review of *Brucella*-host interactions. Am J Pathol. 185(6):1505–1517. doi: 10.1016/j.ajpath.2015.03.003. 25892682 PMC4450313

[CIT0011] De Massis F, Zilli K, Di Donato G, Nuvoloni R, Pelini S, Sacchini L, D’Alterio N, Di Giannatale E. 2019. Distribution of *Brucella* field strains isolated from livestock, wildlife populations, and humans in Italy from 2007 to 2015. PLOS One. 14(3):e0213689. doi: 10.1371/journal.pone.0213689. 30901346 PMC6430384

[CIT0012] DECRETO 2 maggio. 2024. Adozione dei programmi nazionali obbligatori di eradicazione per brucellosi e tubercolosi nei bovini e brucellosi negli ovi-caprini. (24A03318) (GU Serie Generale n.151 del 29-06-2024) [accessed 2025 Mar 17]. https://www.gazzettaufficiale.it/eli/id/2024/06/29/24A03318/SG.

[CIT0013] Dendukuri N, Joseph L. 2001. Bayesian approaches to modeling the conditional dependence between multiple diagnostic tests. Biometrics. 57(1):158–167. doi: 10.1111/j.0006-341x.2001.00158.x. 11252592

[CIT0014] Denwood MJ. 2016. Runjags: an R package providing interface utilities, model templates, parallel computing methods and additional distributions for MCMC models in JAGS. J Stat Soft. 71(9):1–25. doi: 10.18637/jss.v071.i09.

[CIT0015] European Food Safety Authority (EFSA), European Centre for Disease Prevention and Control (ECDC). 2024. The European Union One Health 2023 Zoonoses report. EFSA J. 22:e9106. doi: 10.2903/j.efsa.2024.9106. PMC11629028 39659847

[CIT0016] European Health and Digital Executive Agency (HaDEA). 2023. Brucellosis (*Brucella abortus, B. melitensis and B. suis*) [accessed 2023 Nov 16]. https://hadea.ec.europa.eu/programmes/single-market-programme-food/veterinaryprogrammes/brucellosis_en.

[CIT0017] European Union Reference Laboratory (EURL) for Brucellosis. 2021a. Brucella culture and genus identification standard operating procedure [accessed 2023 Oct 5]. https://sitesv2.anses.fr/sites/default/files/EU%20Brucellosis%20culture%20and%20identification%20SOP%202021%20v4.pdf.

[CIT0018] European Union Reference Laboratory (EURL) for Brucellosis. 2021b. Brucellosis Rose Bengal Test standard operating procedure [accessed 2025 Mar 14]. https://sitesv2.anses.fr/en/minisite/lrue-brucellose/rose-bengal-test.

[CIT0019] European Union Reference Laboratory (EURL) for Brucellosis. 2021c. Brucella typing standard operating procedure. [accessed 2023 Oct 5] https://sitesv2.anses.fr/sites/default/files/EU%20brucellosis_typing%20of%20Brucella%20SOP%202021%20v1.pdf.

[CIT0020] European Union Reference Laboratory (EURL) for Brucellosis. 2021. Brucellosis complement fixation test (EU RL cold and warm incubation) standard operating procedure revision 11. https://sitesv2.anses.fr/en/minisite/lrue-brucellose/complement-fixation-test.

[CIT0021] European Union Reference Laboratory (EURL) for Brucellosis. 2019. Standard Operating Procedure Brucella real time PCR. Version 1 [accessed 2023 Oct 5]. https://sitesv2.anses.fr/sites/default/files/2019%20Brucellosis%20EU-RL%20RT%20PCR.pdf.

[CIT0022] Food and Agriculture Organization of the United Nations. 2013. Regional workshop on Brucellosis control in Central Asia and Eastern Europe. FAO Animal Production and Health Report No. 8. 2015 Rome, Italy [accessed 2023 Nov 6]. https://www.fao.org/3/i4387e/I4387E.pdf.

[CIT0023] G¨urbilek SE, Keskin O, Tel OY. 2023. Development of antigen-capture ELISA using monoclonal antibodies for the detection of Brucellae in milk. Ger J Vet Res. 3(3):1–6. doi: 10.51585/gjvr.2023.3.0056.

[CIT0024] Gall D, Nielsen K. 2004. Serological diagnosis of bovine Brucellosis: a review of test performance and cost comparison. Rev Sci Tech. 23(3):989–1002. 15861895

[CIT0025] Hosein HI, Rouby SR, Menshawy A, AbdAl-Ghany AE. 2017. Sensitivity and specificity of the commonly used diagnostic procedures of bovine brucellosis. Vet Sci Res Rev. 3(3):45–52. doi: 10.17582/journal.vsrr/2017.

[CIT0026] Hui SL, Walter SD. 1980. Estimating the error rates of diagnostic tests. Biometrics. 36(1):167–171. doi: 10.2307/2530508. 7370371

[CIT0027] Hui SL, Zhou XH. 1998. Evaluation of diagnostic tests without gold standards. Stat Methods Med Res. 7(4):354–370. doi: 10.1177/096228029800700404. 9871952

[CIT0028] Johnson WO, Jones G, Gardner IA. 2019. Gold standards are out and Bayes is in: implementing the cure for imperfect reference tests in diagnostic accuracy studies. Prev Vet Med. 167:113–127. doi: 10.1016/j.prevetmed.2019.01.010. 31027713

[CIT0029] Kostoulas P, Nielsen SS, Branscum AJ, Johnson WO, Dendukuri N, Dhand NK, Toft N, Gardner IA. 2017. STARD-BLCM: standards for the reporting of diagnostic accuracy studies that use Bayesian latent class models. Prev Vet Med. 138:37–47. doi: 10.1016/j.prevetmed.2017.01.006. 28237234

[CIT0030] Laine CG, Johnson VE, Scott H, Arenas-Gamboa AM. 2023. Global estimate of human Brucellosis incidence. Emerg Infect Dis. 29(9):1789–1797. doi: 10.3201/eid2909.230052. 37610167 PMC10461652

[CIT0031] Lapraik RD, Moffat R. 1982. Latent bovine Brucellosis. Vet Rec. 111(25–26):578–579. 7157620

[CIT0032] Liu J, Song Z, Ta N, Tian G, Yang X, Zhao H, Piao D, Fan Y, Zhang Y, Jiang H, et al. 2023. Development and evaluation of a droplet digital PCR assay to detect Brucella in human whole blood. PLOS Negl Trop Dis. 17(6):e0011367. doi: 10.1371/journal.pntd.0011367. 37267228 PMC10237378

[CIT0033] Mabe L, Onyiche TE, Thekisoe O, Suleman E. 2022. Accuracy of molecular diagnostic methods for the detection of bovine brucellosis: a systematic review and meta-analysis. Vet World. 15(9):2151–2163. doi: 10.14202/vetworld.2022.2151-2163. 36341063 PMC9631377

[CIT0034] Mahajan V, Banga HS, Filia G, Gupta MP, Gupta K. 2017. Comparison of diagnostic tests for the detection of bovine Brucellosis in the natural cases of abortion. Iran J Vet Res. 18(3):183–189. 29163647 PMC5674441

[CIT0035] Mainar-Jaime RC, Muñoz PM, de Miguel MJ, Grilló MJ, Marín CM, Moriyón I, Blasco JM. 2005. Specificity dependence between serological tests for diagnosing bovine brucellosis in Brucella-free farms showing false positive serological reactions due to *Yersinia enterocolitica* O:9. Can Vet J. 46(10):913–916. 16454384 PMC1255594

[CIT0036] Njeru J, Wareth G, Melzer F, Henning K, Pletz MW, Heller R, Neubauer H. 2016. Systematic review of brucellosis in Kenya: disease frequency in humans and animals and risk factors for human infection. BMC Public Health. 16(1):853. doi: 10.1186/s12889-016-3532-9. 27549329 PMC4994226

[CIT0037] Nyarku R, Hassim A, Jonker A, Quan M. 2020. Development of a genus-specific *Brucella* real-time PCR assay targeting the 16S-23S rDNA internal transcribed spacer from different specimen types. Vet Sci. 7(4):175. doi: 10.3390/vetsci7040175. 33187050 PMC7712849

[CIT0038] O’Grady D, Byrne W, Kelleher P, O’Callaghan H, Kenny K, Heneghan T, Power S, Egan J, Ryan F. 2014. A comparative assessment of culture and serology in the diagnosis of Brucellosis in dairy cattle. Vet J. 199(3):370–375. doi: 10.1016/j.tvjl.2014.01.008. 24507882

[CIT0039] Plummer M. 2023. _rjags: Bayesian graphical models using MCMC_. R package version 4-14. https://CRAN.R-project.org/package=rjags.

[CIT0040] Ramírez-Romero R. 1998. Is *Brucella abortus* a facultative intracellular pathogen with mitochondrialike activity? Med Hypotheses. 51(1):41–45. doi: 10.1016/s0306-9877(98)90252-3. 9881835

[CIT0041] Rodrigues Dos Santos Souza M, Martins Soares Filho P, Arrais Hodon M, Gomes de Souza P, Osório Silva CH. 2022. Evaluation of diagnostic tests’ sensitivity, specificity and predictive values in bovine carcasses showing brucellosis suggestive lesions, condemned by Brazilian Federal Meat Inspection Service in the Amazon Region of Brazil. Prev Vet Med. 200:105567. doi: 10.1016/j.prevetmed.2021.105567. 35016132

[CIT0042] Sistema Informativo Veterinario (VetInfo). [accessed 2024 Jul 11]. https://www.vetinfo.it/j6_rendicontazioniNew/report/ZOB/.

[CIT0043] Tao Z, Chen Q, Chen Y, Li Y, Mu D, Yang H, Yin W. 2021. Epidemiological characteristics of human Brucellosis – China, 2016–2019. China CDC Wkly. 3(6):114–119. doi: 10.46234/ccdcw2021.030. 34595016 PMC8393115

[CIT0044] ter Huurne AA, Meijer M, Dijkerman NA. 1993. De latentie van Brucella abortus veroorzaakt een probleem in de gerichte bestrijding: een overzicht [Latency of *Brucella abortus* causes problems in oriented control: a review]. Tijdschr Diergeneeskd. 118(21):679–683. 8256248

[CIT0045] Tulu D. 2022. Bovine Brucellosis: epidemiology, public health implications, and status of Brucellosis in Ethiopia. Vet Med. 13:21–30. doi: 10.2147/VMRR.S347337. PMC8752066 35028300

[CIT0046] World Organisation for Animal Health (WOAH). Terrestrial manual. 2022. Chapter 3.1.4. Brucellosis (infection with *B. abortus, B. melitensis* and *B. Suis*). Version adopted in 2022. [accessed 2023 Oct 5]. https://www.woah.org/en/what-we-do/standards/codes-and-manuals/#chapter/?rid=300&volume_no=3&ismanual=true&language=102&standard_type=6&animal_type=7

[CIT0047] World Organisation for Animal Health (WOAH). 2023. Terrestrial manual. Chapter 1.1.6. Principles and methods of validation of diagnostic assays for infectious diseases. Version adopted in 2023. https://www.woah.org/fileadmin/Home/fr/Health_standards/tahm/1.01.06_VALIDATION.pdf.

[CIT0048] Zhang N, Huang D, Wu W, Liu J, Liang F, Zhou B, Guan P. 2018. Animal brucellosis control or eradication programs worldwide: a systematic review of experiences and lessons learned. Prev Vet Med. 160:105–115. doi: 10.1016/j.prevetmed.2018.10.002. 30388992

[CIT0049] Zowghi E, Ebadi A, Mohseni B. 1990. Isolation of *Brucella* organisms from the milk of seronegative cows. Rev Sci Tech. 9(4):1175–1178. doi: 10.20506/rst.9.4.525. 2132709

