# Supplementary material for: Large-scale evaluation of bacteriological-based method and qPCR performance for Brucellosis diagnosis in livestock using Bayesian latent class analysis
Source: Vet Q. 2025 Jun 9;45(1):1–10. doi: 10.1080/01652176.2025.2514753 (PMC12150646; doi:10.1080/01652176.2025.2514753)
Supplement: Supplementary_tables.docx [file TVEQ_A_2514753_SM4573.docx]

**Supplementary table 1.** STARD-BLCM checklist. The table displays the adherence to guidelines for reporting on study design, conduct and results of diagnostic accuracy.

| **Section & Topic** | **Item** | **STARD-BLCM** | **Page** |
| --- | --- | --- | --- |
| Title/Abstract/Keywords | | | |
|  | 1 | Identification as a study of diagnostic accuracy, using at least one measure of accuracy (such as sensitivity, specificity, predictive values, or AUC) and Bayesian latent class models | Yes |
| Abstract | | | |
|  | 2 | Structured summary of study design, methods, results, and conclusions (for specific guidance, see STARD for Abstracts) | Yes |
| Introduction | | | |
|  | 3 | Scientific and clinical background, including the intended use and clinical role of the tests under evaluation | Yes |
|  | 4 | Study objectives and hypotheses, such as estimation of diagnostic accuracy of the tests for a defined purpose through BLCM | Yes |
| Methods | | | |
| *Study design* | 5 | Whether data collection was planned before the tests were performed (prospective study) or after (retrospective study) | Yes |
| *Participants* | 6 | Eligibility criteria and description of the source population | Yes |
|  | 7 | On what basis potentially eligible participants were identified (such as symptoms, results from previous tests, inclusion in registry) | Yes |
|  | 8 | Where and when potentially eligible participants were identified (setting, location, and dates) | Yes |
|  | 9 | Whether participants formed a consecutive, random or convenience series | Yes |
| *Test methods* | 10 | Description of the tests under evaluation, in sufficient detail to allow replication, and/or cite references | Yes |
|  | 11 | Rationale for choosing the tests under evaluation in relation to their purpose | Yes |
|  | 12 | Rationale for test positivity cut-offs or result categories of the tests under evaluation, distinguishing pre-specified from exploratory | From reference |
|  | 13 | Whether clinical information was available to the performers or readers of the tests under evaluation | Not applicable |
| *Analysis* | 14a | BLCM model for estimating measures of diagnostic accuracy | Yes |
|  | 14b | Definition and rationale of prior information and sensitivity analysis | Yes |
|  | 15 | How indeterminate results of the tests under evaluation were handled | Not applicable |
|  | 16 | How missing data of the tests under evaluation were handled | Not applicable |
|  | 17 | Any analyses of variability in diagnostic accuracy, distinguishing pre-specified from exploratory | Yes |
|  | 18 | Intended sample size and how it was determined | Not applicable |
| Results | | | |
| *Participants* | 19 | Flow of participants, using a diagram | Yes, no diagram |
|  | 20 | Baseline demographic and clinical characteristics of participants | No |
|  | 21 | The distribution of the targeted conditions is unknown, hence the use of BLCM | Yes |
|  | 22 | Time interval and any clinical interventions between the tests under evaluation | Not applicable |
| *Test results* | 23 | Cross tabulation of the tests’ results (or for continuous tests results their distribution by infection stage) | Yes |
|  | 24 | Estimates of diagnostic accuracy under alternative prior specification and their precision (such as 95% credible/probability intervals) | Yes |
|  | 25 | Report any adverse events from performing the of the tests under evaluation | Yes |
| Discussion | | | |
|  | 26 | Study limitations, including sources of potential bias, statistical uncertainty, and generalisability | Yes |
|  | 27 | Implications for practice, including the intended use and clinical role of the tests under evaluation in relevant settings (clinical, research, surveillance etc.) | Yes |
| Other information | | | |
|  | 28 | Registration number and name of registry | No |
|  | 29 | Where the full study protocol can be accessed | No |
|  | 30 | Sources of funding and other support; role of funders | Yes |

**Supplementary table 2.** Results of direct tests for *Brucella* spp used in series and in parallel. The table displays the results of bacteriological culture and qPCR as they were used in series or in parallel to classify seronegative animals.

| **Seronegative animals** | | **Bacteriological culture AND qPCR** | |  |
| --- | --- | --- | --- | --- |
|  |  | **−** | **+** | **Total (%)** |
| **Bacteriological culture OR qPCR** | **−** | 993 |  | 993 (75.7) |
|  | **+** | 238 | 80 | 318 (24.3) |
|  | **Total (%)** | 1,231 (93.9) | 80 (6.1) | 1,311 |

**Supplementary table 3.** Results of direct tests for *Brucella* spp used in series and in parallel. The table displays the results of bacteriological culture and qPCR as they were used in series or in parallel to classify seropositive animals.

| **Seropositive animals** | | **Bacteriological culture AND qPCR** | |  |
| --- | --- | --- | --- | --- |
|  |  | **−** | **+** | **Total (%)** |
| **Bacteriological culture OR qPCR** | **−** | 2,287 |  | 2,287 (59.6) |
|  | **+** | 1,130 | 421 | 1,551 (40.4) |
|  | **Total (%)** | 3,417 (89.0) | 421 (11.0) | 3,838 |

**Supplementary table 4.** Model diagnostics parameters. The table displays Monte Carlo error (MCerr) and its percentage of standard deviation (%ofSD), effective sample size (SSeff), autocorrelation at lag 10 (AC10), potential scalar reduction factor or Gelman-Rubin diagnostics ($\hat{R}$), and the mean of simualted data compared to that of observed data.

| **Parameter** | **MC_err_**  **(% of SD)** | **SS_eff_** | **AC100** | $\hat{R}$ | **Avg_simulated_** | **Avg_observed_** |
| --- | --- | --- | --- | --- | --- | --- |
| Se_bacteriol_ | 0.0001 (0.5) | 37,447 | -0.0021 | 1.0000 | - | - |
| Se_qPCR_ | 0.0001 (0.5) | 39,909 | -0.0054 | 1.0000 | - | - |
| Sp_bacteriol_ | 0.0000 (0.6) | 32,431 | 0.0017 | 1.0000 | - | - |
| Sp_qPCR_ | 0.0001 (0.5) | 34,601 | -0.0033 | 1.0000 | - | - |
| Prev_pop1_ | 0.0001 (0.5) | 38,799 | 0.0043 | 1.0000 | 0.0127 | 0.0160 |
| Prev_pop2_ | 0.0001 (0.6) | 24,605 | 0.0079 | 1.0000 | 0.0465 | 0.1282 |
| Prev_pop3_ | 0.0007 (0.6) | 26,946 | 0.0060 | 1.0000 | 0.2000 | 0.3525 |
| Prev_pop4_ | 0.0005 (1.0) | 10,468 | 0.0660 | 1.0001 | 0.1778 | 0.1978 |
| cov_Se_ | 0.0000 (0.5) | 37,180 | 0.0088 | 1.0000 | - | - |
| cov_Sp_ | 0.0000 (0.5) | 38,780 | -0.0007 | 1.0001 | - | - |
